# Lycopene presence in facial skin corneocytes and sebum and its association with circulating lycopene isomer profile: Effects of age and dietary supplementation

**DOI:** 10.1002/fsn3.799

**Published:** 2019-03-13

**Authors:** Ivan M. Petyaev, Dmitry V. Pristensky, Elena Y. Morgunova, Nailya A. Zigangirova, Valeriy V. Tsibezov, Natalia E. Chalyk, Victor A. Klochkov, Victoria V. Blinova, Tatiana M. Bogdanova, Alexei A. Iljin, Larisa S. Sulkovskaya, Marina P. Chernyshova, Marina V. Lozbiakova, Nigel H. Kyle, Yuriy K. Bashmakov

**Affiliations:** ^1^ Lycotec Ltd Cambridge UK; ^2^ Gamaleya Research Institute of Epidemiology and Microbiology Moscow Russia; ^3^ Saratov State Medical University Research Institute of Cardiology Saratov Russia

**Keywords:** aging, antioxidant carotenoid, dietary supplementation, serum lycopene isomers, skin surface

## Abstract

Lycopene is a dietary antioxidant known to prevent skin photodamage. This study aimed to examine age‐dependent presence of this carotenoid on the surface of the facial skin and in the serum as well as to measure the same parameters during supplementation with lycopene. Serum samples and samples from facial skin surface were obtained from 60 young (under 25 years old) and 60 middle‐aged (over 50 years old) volunteers. Similar samples were taken from 15 middle‐aged subjects during 4‐week supplementation with lycopene (7 mg/day). Serum lycopene levels and isomer profiles were analyzed by HPLC. Lycopene in desquamated corneocytes and the sebum from facial skin surface was determined using lycopene‐specific fluorescent monoclonal antibodies. The results demonstrated that there was no age‐related difference in serum lycopene levels, but a higher proportion of (all‐E)‐lycopene was detected in the “young” group (37.5% vs 26.2% in the “middle‐aged” group; *p *<* *0.0001). “Young” volunteers also had a higher lycopene level in both corneocytes (*p *=* *0.0071) and the sebum (*p *=* *0.0139) from the skin surface. Supplementation with lycopene resulted in a sharp increase of lycopene concentrations in both serum and skin surface samples. There was also a clear change in the pattern of lycopene isomers in the serum manifested by a significant increase in the proportion of (all‐E)‐lycopene (from 22.1% to 44.0% after supplementation, *p *<* *0.0001). It can be concluded that dietary supplementation with lycopene results in its accumulation in the serum and skin. This process is accompanied by significant changes in the circulating lycopene isomer profile which becomes similar to that typical for young individuals.

## 1 INTRODUCTION

Carotenoids constitute a large group of plant pigments possessing strong antioxidant properties that are routinely ingested by humans with fruit and vegetables (Kaulmann & Bohn, 2014; Rao & Rao, 2007). Lycopene, a hydrocarbon carotenoid abundantly present in tomatoes, has recently attracted considerable attention as a common dietary factor, consumption of which is associated with a decreased risk of major chronic diseases (cardiovascular disease, cancer) and antiaging effects (Petyaev, 2016; Rao & Rao, 2007; Story, Kopec, Schwartz, & Harris, 2010). One of the actively investigated beneficial effects of lycopene is its photoprotective action against UV‐induced damage to human skin (Gretner‐Beck, Marini, Jaenicke, Stahl, & Krutmann, 2017; Rizwan et al., 2011; Stahl & Sies, 2012) that may also contribute to skin aging (Jenkins, Wainwright, Holland, Barrett, & Casey, 2014). The mechanisms behind these phenomena are not entirely clear, being currently explained by antioxidant action of the carotenoid (Gretner‐Beck et al., 2017; Jenkins et al., 2014; Rizwan et al., 2011; Stahl & Sies, 2012). Lycopene concentration in the skin correlates with its level in the plasma (Scarmo et al., 2010), and accumulation of this carotenoid in the skin during dietary supplementation is well documented (Blume‐Peytavi et al., 2009; Meinke, Darvin, Vollert, & Lademann, 2010; Ross et al., 2011; Walfisch, Walfisch, Agbaria, Levy, & Sharoni, 2003). Lycopene concentration is usually measured using HPLC that can be applied to skin biopsy samples (Scarmo et al., 2010; Walfisch et al., 2003). Biopsy taking is, however, invasive and unsuitable for assessing facial skin. Although resonance Raman spectroscopy was proposed as a feasible method for noninvasive carotenoid quantification in this location (Blume‐Peytavi et al., 2009; Ermakov, Ermakova, Gellermann, & Lademann, 2004; Mayne et al., 2010), the use of this technique requires expensive equipment that may not be easily available. For this reason, simpler alternative methods of noninvasive detection of lycopene and other carotenoids may be welcome.

Our group has generated a novel monoclonal antibody against lycopene that was demonstrated to be suitable for immunofluorescent recognition of this carotenoid in both cultured cells and material collected from the surface of human facial skin (Tsibezov et al., 2017). We decided to combine this new detection method with our recently described technique for noninvasive sampling of material rich in sebum and desquamated corneocytes from the surface of human facial skin (Chalyk, Bandaletova, Kyle, & Petyaev, 2017). In this study, we assessed feasibility of applying this approach to analyzing samples collected from skin surface of volunteers of different age in parallel with determining lycopene concentrations and lycopene isomer profiles in serum samples from the same subjects. In addition, lycopene accumulation in the serum and on the surface of the facial skin was analyzed in a subgroup of middle‐aged volunteers during dietary supplementation with lycopene for 4 weeks.

## 2 MATERIALS AND METHODS

### 2.1 Subjects

This study was conducted by Lycotec Ltd (Cambridge, UK) in collaboration with the Institute of Cardiology of the Ministry of Health of the Russian Federation (Saratov, Russian Federation) and the Gamaleya Research Institute of Epidemiology and Microbiology (Moscow, Russian Federation). The protocol of the study was approved by the local Ethics Committee (FGBU SarNIIK 18.02.2014) confirming that the study conformed to the European Medicines Agency Guidelines for Good Clinical Practice.

All study participants were informed about the purpose of the investigation and provided written informed consent. This study is a part of a larger project registered as ISRCTN89815519 in the ISRCTN registry.

Study participants were recruited in Saratov, from the existing pool of healthy volunteers. The main inclusion criteria corresponded with the target of forming two age‐defined groups of clinically healthy Caucasian males and females: under 25 years old (“young”) and over 50 years old (“middle‐aged”). All recruited volunteers were free of systemic chronic disorders, conditions affecting skin, and food allergies.

### 2.2 Study design

The total number of volunteers recruited to take part in the study was 120 (60 men and 60 women). Two age groups were formed as follows: 60 volunteers (30 men and 30 women) under 25 years of age (“young” group) and 60 volunteers (30 men and 30 women) over 50 years of age (“middle‐aged” group).

In addition, 15 volunteers from the “middle‐aged” group (seven men and eight women) took part in a substudy where they received daily 7 mg doses of lycopene (GA Lycopene capsules manufactured by Lycotec Ltd, Cambridge, UK) as a dietary supplement for 4 weeks. The study was conducted in February–March, when dietary lycopene intake in Saratov is at its lowest.

Once recruited, study participants had their body mass index (BMI) determined by measuring body mass and height in the morning and then calculating the index in kg/m^2^. Individuals with BMI below 18.5 kg/m^2^ were regarded as underweight; subjects with normal weight were in the range between 18.5 and 25 kg/m^2^, those with BMI in the range between 25 and 30 kg/m^2^ were classified as overweight, and BMI values over 30 kg/m^2^ indicated obesity.

### 2.3 Sample collection and preparation

Blood samples were collected by phlebotomy from all study participants in the morning after night fast. In the substudy with lycopene supplementation, additional blood collections were performed after 2 weeks (day 15) and 4 weeks (day 29) of lycopene consumption. The serum was separated by centrifugation, aliquoted, placed in code‐labelled tubes for blinded analysis, and stored at −80°C until use.

For sample collection from the surface of the facial skin, all study participants were requested to avoid facial hygienic manipulations for 24 hr before sampling, which was carried out in the morning in parallel with blood sample collection. Skin surface sample collection and preparation was performed as previously described (Chalyk et al., 2017). Briefly, samples were collected using polyester swabs from the surface of the facial skin (the sides of the nose). During the procedure, two samples were taken (one swab per side). Each collected sample was placed on the surface of a microscope slide. A second microscope slide was pressed against the surface of the first one. This procedure provided a pair of identical smears. The smears were thoroughly dried.

All slides with collected samples were coded to provide sample anonymity for blinded analysis and stored at −20°C until use.

### 2.4 Lycopene quantification in serum samples

Lycopene concentration and isomer ratio measurement in all serum samples were carried out using high‐performance liquid chromatography (Diwadkar‐Navsariwala et al., 2003) with modifications. Briefly, 400 μl of serum was mixed with 400 μl of ethanol and extracted twice with 2 ml hexane. The combined hexane layers were evaporated to dryness under vacuum (Scan Speed 32 centrifuge) and the residue reconstituted to 100 μl in sample solution (absolute ethanol–methylene chloride, 5:1, v/v). The specimens were centrifuged again (15 min at 10,000 *g*), and the supernatant was transferred to HPLC vials. The extract (5 μl) was injected into an Acquity HSS T3 75 × 2.1 mm 1.8 μm column (Waters, USA) preceded by a Acquity HSS T3 1.8 μm VanGuard precolumn (Waters, USA) and eluted isocratically at 45°C with the mobile phase (acetonitrile—0.08% phosphoric acid solution—tert‐butyl methyl ether, 70:5:25, v/v/v) at a flow rate of 0.5 ml/min. The peaks corresponding to lycopene isomers were detected by a Photodiode Array Detector (Waters, USA) at 474 nm. The peak areas were measured using Empower 3 software (Waters, MA). Lycopene concentrations in serum samples were calculated by reference to an analytical standard (lycopene from tomato, L9879, Sigma, USA). Relative concentrations of (all E) and (Z)‐lycopene isomers were calculated by comparing their peak areas to the standard curve as previously described (Fang, Pajkovic, Wang, Gu, & van Breemen, 2003).

### 2.5 Lycopene detection in facial sebum and desquamated corneocytes by immunofluorescent staining

**Figure 1:**
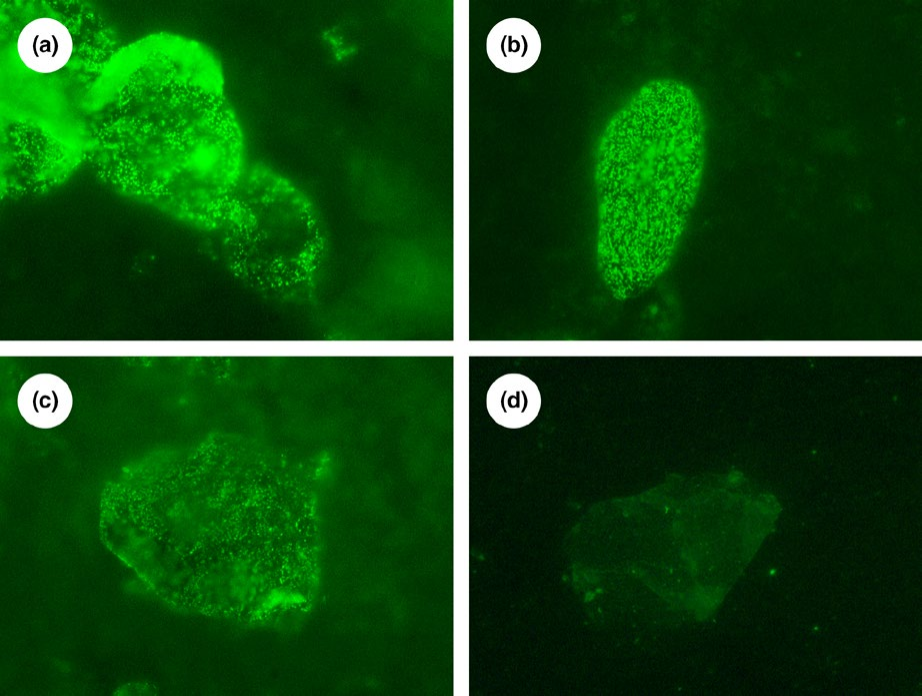
Photomicrographs of samples noninvasively collected from the surface of the facial skin and stained using our immunofluorescent technique (magnification ×1,000 in all cases) exemplifying fluorescence scoring system used in the study: (a) Extremely strong fluorescence of corneocytes (score = 4) and weak fluorescence of sebum background (score = 1), (b) Strong fluorescence of a corneocyte (score = 3) and fluorescence traces in sebum background (score = 0.5), (c) Moderate fluorescence of a corneocyte (score = 2) and moderate fluorescence of sebum background (score = 2), (d) Weak fluorescence of a corneocyte (score = 1) and no fluorescence of sebum background (score = 0)

Dried smears collected from the surface of the facial skin were used for immunofluorescent staining. Desquamated corneocytes and sebum present in the material were stained for direct immunofluorescence using fluorescein isothiocyanate‐conjugated monoclonal antibody against lycopene recently generated by our group (Tsibezov et al., 2017). Fluorescent staining was visualized using Nikon Eclipse 50i microscope with a fluorescence attachment. The semiquantitative analysis was based on visually assessing fluorescence levels in corneocytes and surrounding sebum in 20 random fields of view at ×200 magnification. Fluorescence intensity in the samples was classified using the following scoring system illustrated in Figure 1: 0—no fluorescence; 0.5—traces of fluorescence; 1—weak fluorescence; 2—moderate fluorescence; 3—strong fluorescence of some cells or areas of sebum background; 4—extremely strong fluorescence (confluent fluorescent elements within corneocytes). Fluorescence assessment in each sample was repeated blindly three times.

### 2.6 Data analysis

All quantitative results for comparing “young” and “middle‐aged” volunteers were calculated for each of the two groups and for male and female subjects separately. In the substudy with lycopene supplementation, results were calculated for the three sampling time points (before supplementation, at 2 weeks, and at 4 weeks).

Results of all quantitative measurements (or fluorescence score counts) were analyzed using descriptive statistics (mean, standard deviation, median and range values as well as 95% confidence intervals were determined). Paired t test (two‐sided *p*‐values calculated) was applied to determine statistical significance for the differences between time points. *t* test for independent means was used for comparing groups. Pie charts were employed for presenting proportions of lycopene isomers in the serum for the age groups and for different time points of the supplementation substudy. All data handling and statistical analyses were performed using IBM SPSS 19.0 statistical package (IBM Inc., Armonk, NY, USA).

## 3 RESULTS

### 3.1 General characteristics of the study population

Age distributions in the two study groups were relatively compact. In “young” volunteers, the mean age was 18.7 (95% CI: 18.2–19.1) with similar distributions among male and female study participants. In the “middle‐aged” group, the mean age was 65.0 (95% CI: 63.4–66.5) without gender‐related differences. There was an obvious difference between the groups in BMI values. The mean BMI in the “young” group was 22.3 (95% CI: 21.3–23.2), which corresponds to normal weight. Among 60 study participants of this group, 45 (75%) were classified as normal, five (8.3%) as underweight, seven (11.7%) as overweight, and only three (5%) as obese. In contrast, in the “middle‐aged” group, the mean BMI was 29.4 (95% CI: 28.3–30.5), corresponding to overweight status. Only nine (15%) members of this group had normal weight, whereas 26 (43.3%) volunteers were classified as overweight and 25 (41.7%) were obese.

### 3.2 Lycopene measurement in the serum and material collected from the surface of the skin in “young” and “middle‐aged” groups of volunteers

Table 1 shows that serum lycopene concentrations in both “young” and “middle‐aged” volunteers were generally low. Among 120 study participants, only 37 had serum lycopene levels above 300 nM. In particular, low lycopene concentrations were observed in “young” women. The mean serum lycopene value in this subgroup (139.70 nM) was significantly lower than in either “young” men or “middle‐aged” women (Table 1).

**Table 1:** Lycopene measurement in the serum and material from the surface of the skin of volunteers of different age groups

Variables	Young volunteers			Middle‐aged volunteers		
	Whole group	Females	Males	Whole group	Females	Males
Number	60	30	30	60	30	30
Serum samples						
Lycopene, nM	223.5 (183.1–263.9)	139.7^a,b^ (110.5–168.9)	307.3^a^ (243.3–371.4)	290.6 (234.3–346.8)	281.3^b^ (216.6–345.9)	299.9 (203.2–396.5)
Lycopene isomers (serum)						
% of (all‐E)	37.5^c^ (35.7–39.3)	35.1^d^ (3.72–37.5)	39.9^e^ (37.5–42.4)	26.2^c^ (24.4–28.0)	24.6^d^ (22.1–27.1)	27.9^e^ (25.3–30.4)
% of (5Z)	27.2^f^ (25. 8–28.7)	27.1^g^ (25.4–28.8)	27.3 (24.9–29.8)	22.0^f^ (20.5–23.5)	19.5^g,h^ (18.0–21.1)	24.5^h^ (22.1–26.9)
% of (9Z)	11.2^i^ (10.4–12.1)	12.4^j^ (11.3–13.6)	10.0^j,k^ (8.8–11.2)	13.9^i^ (12.8–15.1)	13.9 (12.2–15.6)	13.9^k^ (12.3–15.6)
% of (13Z)	19.1^l^ (17.3–21.1)	19.7^m^ (16.4–23.1)	18.6^n,o^ (16.6–20.6)	31.5^l^ (29.0–34.1)	34.7^m,o^ (31.3–38.0)	28.4^n,o^ (24.6–32.1)
% of (15Z)	4.7^p^ (4.1–5.4)	5.4^q^ (4.6–6.1)	4.1^r^ (3.1–5.1)	6.3^p^ (5.7–6.9)	7.3^q^ (6.4–8.2)	5.3^r^ (4.5–6.1)
Material from skin surface						
Lycopene in corneocytes, score units	1.53^s^ (1.27–1.80)	1.75^t^ (1.35–2.14)	1.33 (0.97–1.69)	1.02^s^ (0.75–1.28)	0.87^t^ (0.44–1.30)	1.17 (0.83–1.51)
Lycopene in the sebum, score units	0.77^u^ (0.68–0.85)	0.75 (0.62–0.88)	0.78 (0.66–0.91)	0.59^u^ (0.43–0.75)	0.56 (0.39–0.74)	0.62 (0.46–0.77)

*Note*

^a^/*p* < 0.0001; ^b^/*p* = 0.0001; ^c^/*p* < 0.0001; ^d^/*p* < 0.0001; ^e^/*p* < 0.0001; ^f^/*p* < 0.0001; ^g^/*p* < 0.0001; ^h^/*p* = 0.0006; ^i^/*p* = 0.0003; ^j^/*p* = 0.0031; ^k^/*p* = 0.0002; ^l^/*p* < 0.0001; ^m^/*p* < 0.0001; ^n^/*p* < 0.0001; ^o^/*p* = 0.0126; ^p^/*p* = 0.0008; ^q^/*p* = 0.0017; ^r^/*p* = 0.0016; ^s^/*p* = 0.0071; ^t^/*p* = 0.0035; ^u^/*p* = 0.0139.

Values are given as means (95% CI).

**Figure 2:**
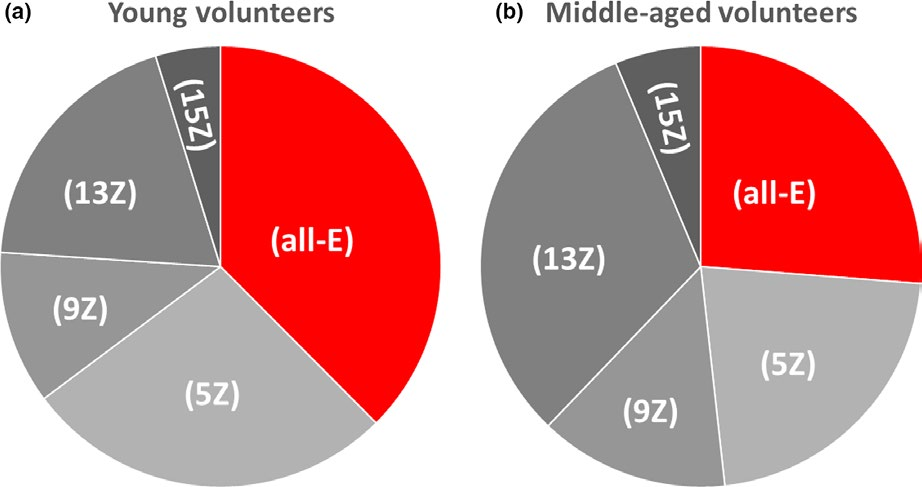
Pie charts showing lycopene isomer proportions in the serum of “young” (a) and “middle‐aged” volunteers (b). See Table 1 for precise results

An obvious difference between the age‐defined groups was observed in the proportions of lycopene isomers (Table 1 and Figure 2). Among “middle‐aged” volunteers, the proportion was significantly shifted toward *cis*‐forms, especially because of the increase in the share of *13‐cis*‐lycopene which accounted for 31.52% of all serum lycopene in this group.

Semiquantitative assessment of lycopene amount in the material collected from the surface of the skin indicated that lycopene presence in “young” subjects was significantly higher than in the “middle‐aged” group. This difference was observed for both desquamated corneocytes and surrounding sebum.

### 3.3 Lycopene measurement in the serum and material collected from the surface of the skin during dietary supplementation with lycopene

**Figure 3:**
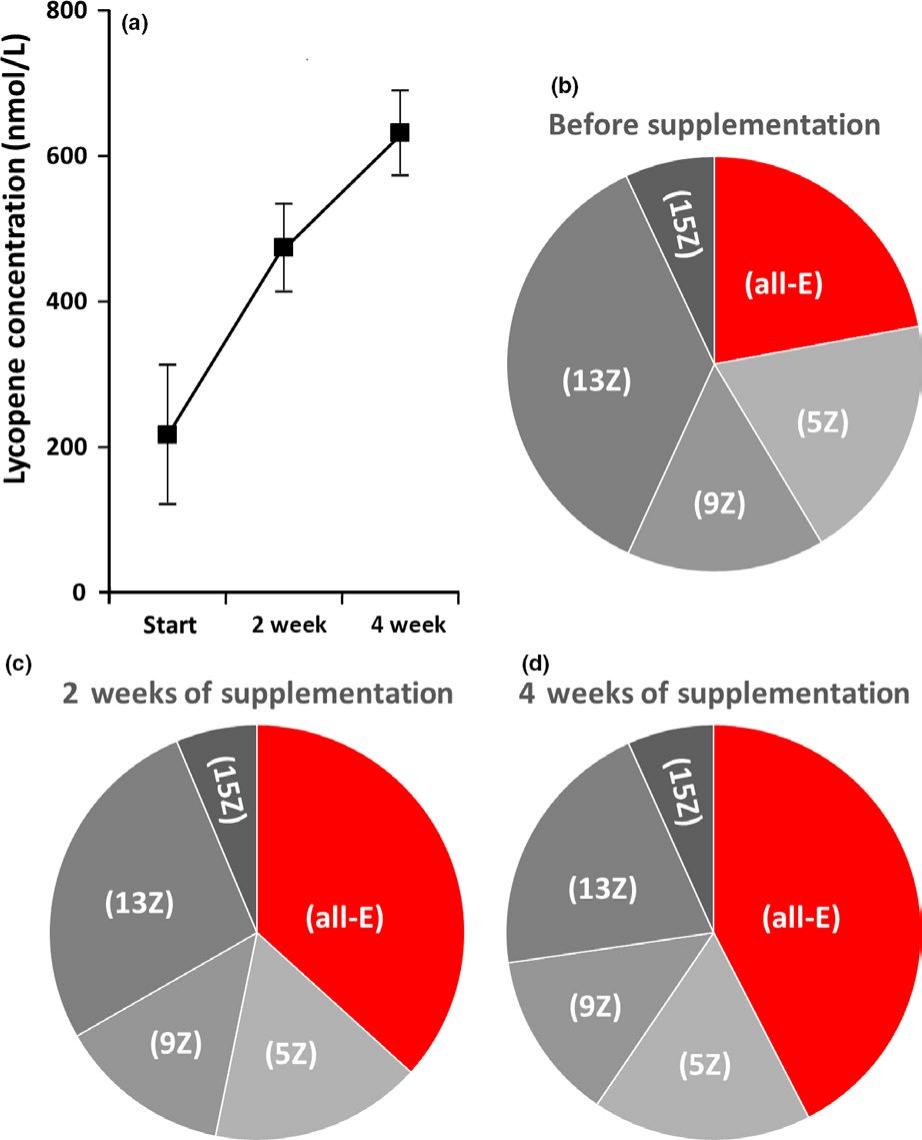
Results of serum lycopene analysis during lycopene supplementation: (a) Changes in serum lycopene concentration (Means and 95% CI), (b) Pie chart showing lycopene isomer proportion before supplementation (see Table 2 for precise results), (c) Pie chart showing lycopene isomer proportion in the middle (2 weeks) of the supplementation period (see Table 2 for precise results), (d) Pie chart showing lycopene isomer proportion at the end (4 weeks) of the supplementation period (see Table 2 for precise results)

**Figure 4:**
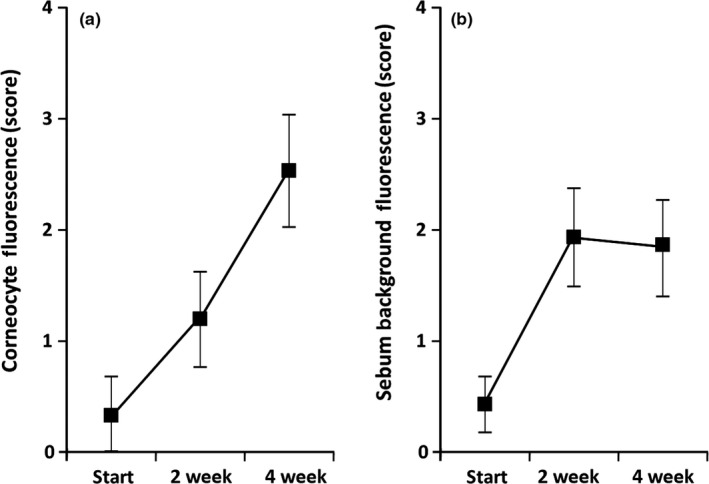
Results of lycopene analysis in samples obtained from facial skin surface: (a) Changes of lycopene level in desquamated corneocytes (Means and 95% CI), (b) Changes of lycopene level in the sebum (Means and 95% CI)

Results of the substudy that included 15 “middle‐aged” volunteers receiving dietary supplementation with lycopene for 4 weeks are presented in Table 2 and Figures 3 and 4. Although general characteristics of the participants of the substudy (Mean age = 66.1; Mean BMI = 29.5) did not differ from the whole “middle‐aged” group (see Table 1), it should be noted that in terms of body composition, nine participants in this substudy were classified as obese, four as overweight, and only two as normal.

**Table 2:** Lycopene measurement in the serum and material from the surface of the skin of 15 volunteers at different time points of dietary supplementation

Variables	Time point		
	Start	2 weeks	4 weeks
Serum samples			
Lycopene, nM	217.3^a,b^ (121.6–313.2)	474.4^a,c^ (413.9–535.0)	632.1^b,c^ (547.1–690.1)
Lycopene isomers (serum)			
% of (all‐E)	22.1^d,e^ (19.2–25.0)	36.7^d,f^ (32.1–41.3)	44.0^e,f^ (40.5–47.6)
% of (5Z)	19.3^g,h^ (17.2–21.4)	16.5^g,i^ (14.9–18.0)	14.7^h,i^ (13.1–16.3)
% of (9Z)	15.4^j^ (12.9–18.0)	13.5^j^ (10.8–16.2)	13.7 (11.5–15.8)
% of (13Z)	36.2^k,l^ (31.1–41.3)	27.0^k,m^ (23.4–30.5)	21.4^l,m^ (19.1–23.7)
% of (15Z)	7.0 (5.3–8.7)	6.3 (5.0–7.6)	6.2 (5.0–7.3)
Material from skin surface			
Lycopene in corneocytes, score units	0.33^n,o^ (0.00–0.68)	1.20^n,p^ (0.77–1.63)	2.53^o,p^ (2.03–3.04)
Lycopene in the sebum, score units	0.43^q,r^ (0.18–0.69)	1.93^q^ (1.49–2.38)	1.87^r^ (1.40–2.33)

*Note*

^a^/*p* < 0.0001; ^b^/*p* < 0.0001; ^c^/*p* = 0.0003; ^d^/*p* < 0.0001; ^e^/*p* < 0.0001; ^f^/*p* < 0.0001; ^g^/*p* = 0.0131; ^h^/*p* = 0.0005; ^i^/*p* = 0.0011; ^j^/*p* = 0.0126; ^k^/*p* < 0.0001; ^l^/*p* < 0.0001; ^m^/*p* < 0.0001; ^n^/*p* = 0.0001; ^o^/*p* = 0.0013; ^p^/*p* < 0.0001; ^q^/*p* < 0.0001; ^r^/*p* < 0.0001.

Values are given as means (95% CI).

Table 2 and Figure 3a clearly demonstrate that serum lycopene concentration was significantly increasing throughout the supplementation period reaching a mean value of 632.07 nM by the end of the study. In parallel, changes in the proportion of lycopene isomers were observed (Figure 3b–d), and these changes followed a clear pattern characterized by a significant increase in the proportion of (all‐E)‐lycopene accompanied by a relative decrease in (13Z)‐lycopene. While the profile of lycopene isomers in the 15 participants of the substudy before supplementation (Figure 3b) did not differ from that of the whole “middle‐aged” group (Figure 2b), after 2 weeks of supplementation (Figure 3c), it became close to the pattern observed in the “young” group (Figure 2a) and continued progressing further by the end of the study (Figure 3d).

Gradual lycopene accumulation during supplementation was also evident in the material collected from the surface of the facial skin (Figure 4). However, there was a difference between desquamated corneocytes and unstructured sebum. Lycopene presence in desquamated corneocytes significantly increased for the whole period of supplementation (Figure 4a). In contrast, sebum assessment revealed lycopene presence increasing only during the first 2 weeks of supplementation. No further lycopene concentration increase in the sebum was observed by the end of the study (Figure 4b).

## 4 DISCUSSION

Parallel increase of lycopene concentrations in the serum and skin during consumption of lycopene‐rich diets or dietary supplementation is well‐documented (Meinke et al., 2010; Mayne et al., 2010; Scarmo et al., 2010; Walfisch et al., 2003), and our results indicating a steep increase in serum lycopene throughout the supplementation period are not surprising. It should be noted that serum lycopene concentrations in the participants of this study were considerably lower than those reported by others (Allen et al., 2003; Gamji & Kafai, 2005; Scarmo et al., 2010) and corresponded to a state of lycopene deficiency (Mackinnon, Rao, & Rao, 2011). This observation apparently reflects the seasonal decrease in dietary lycopene consumption patterns still common in some regions characterized by long winter periods. Nevertheless, the background of lycopene deficiency among study participants helped facilitate the detection of lycopene supplementation effects.

Our analysis of lycopene isomer profiles in serum samples from young and middle‐aged volunteers has resulted in one unexpected finding demonstrating an obvious difference in isomer proportions between the groups. Lycopene is present in dietary sources almost exclusively in the linear (all‐E) conformation, but following ingestion tends to be partially transformed to the (Z)‐forms that are deemed to be more bioavailable (Boileau, Boileau, & Erdman, 2002). In our study, the proportion of (all‐E)‐lycopene was significantly higher among young subjects, whereas middle‐aged volunteers had the highest proportion of (13Z)‐lycopene (Figure 2b). It should be noted that the latter isomer is a major early product of lycopene degradation (Graham, Carail, Caris‐Veyrat, & Lowe, 2012). The difference between our age‐defined study groups in lycopene isomer patterns is clear, but its interpretation needs to be careful. Although it may seem that the age of volunteers is the main defining factor, the two groups were also characterized by a significant difference in body composition. Young volunteers typically were of normal weight, whereas most of those in the “middle‐aged” group were either overweight or obese. Therefore, the difference in lycopene isomer profiles could also be attributed to the influence of body composition.

It is interesting that the dynamics of lycopene isomer proportion changes during supplementation with capsules containing almost exclusively (all‐E)‐lycopene corresponded to a gradual replacement of the “middle‐aged” pattern with the “young” one. Low amplitude changes of the (E)/(Z) ratio in parallel to lycopene withdrawal or supplementation have been described previously, but without specifying different (Z)‐variants (Allen et al., 2003). In our study, we observed significant growth in the proportion of (all‐E)‐lycopene at the expense of (Z)‐lycopene, especially its (13‐Z)‐form (Figure 3). Given the parallel increase of the overall lycopene concentration in the serum, it can be assumed that absolute amounts of all isomers should be increasing, and isomer pattern changes may reflect partial saturation of some metabolic pathways possibly generating biologically active derivatives of lycopene, such as apo‐10′‐lycopenoids (Wang, 2012).

It may be tempting to conclude from lycopene isomer profile changes that lycopene supplementation produces a rejuvenating effect in “middle‐aged” subjects; however, it is equally likely that the dietary intervention may simply restore the normal pattern in overweight or obese individuals. In any case, the effect appears to be unconditionally beneficial. This conclusion is supported by reports on mediation of cardiovascular disease risk biomarkers by serum carotenoids (Kim et al., 2011; Wang et al., 2014) and even association between higher serum lycopene levels and reduced mortality in individuals with metabolic syndrome (Han, Meza, Soliman, Islam, & Watanabe‐Galloway, 2016). Regarding the skin, it can be manifested by increasing protection against UV (Gretner‐Beck et al., 2017; Rizwan et al., 2011; Stahl & Sies, 2012) and antiaging effect (Jenkins et al., 2014).

The stratum corneum (SC) of the epidermis is the most superficial layer of the skin and exerts key barrier functions protecting the human body from adverse environmental influences (Menon, Cleary, & Lane, 2012; Proksch, Brandner, & Jensen, 2008). It is important to emphasize that investigation by Raman spectrometry showed that the upper layers of the SC had the highest concentration of carotenoids in the skin (Darvin et al., 2009), but the authors of the report noted that it was technically difficult to use Raman spectrometry for analyzing the skin surface (Darvin et al., 2009). We believe that our new approach employing recently generated antilycopene antibodies (Tsibezov et al., 2017) presents an attractive alternative. The study described here convincingly demonstrates the feasibility of applying an immunofluorescent technique for detecting lycopene in the material collected from the surface of human facial skin. This material in fact corresponds to the residual skin surface components (RSSC) (Shetage et al., 2014), noninvasive collection and morphological analysis of which is described in our recent paper (Chalyk et al., 2017). Although it can be admitted that the semiquantitative method of fluorescence intensity assessment used in the current study may not be ideal, the results look convincing. Lycopene concentrations in both desquamated corneocytes and sebum were significantly higher in young volunteers. This finding corroborates previous observations of age‐associated decrease of lipophilic antioxidants in the sebum (Passi, De Pita, Puddu, & Littarru, 2002) and probably reflects the development of aging‐related deficiency in skin barrier functions (Boireau‐Adamezyk, Baillet‐Guffroy, & Stamatas, 2014) including antioxidant transport to the skin.

Our substudy with lycopene supplementation showed different patterns of lycopene accumulation in desquamated corneocytes and surrounding sebum (Figure 4). This difference appears to be easy to explain by the physiological necessity of the transit of terminally differentiated corneocytes through the SC that takes approximately 14 days (Haftek, 2014). Therefore, desquamation of the first corneocytes enriched with lycopene should start only by the end of the second week of supplementation and is likely to be fully manifested later as we observe (Figure 4a). In contrast, the level of lycopene in the sebum appears to already reach a “plateau” after 2 weeks of supplementation.

Unfortunately, we were not able to assess relative amounts of lycopene isomers in the material collected from the skin surface. Nevertheless, it is interesting to note that some authors believe that the presence of (Z)‐lycopene in the skin provides increased UV absorption (Stahl & Sies, 2012), thus preventing UV‐induced damage.

## 5 CONCLUSION

Taken together, our results for the skin and serum complement each other and can be interpreted as manifestations of either the “normalizing” or “rejuvenating” action of lycopene in “middle‐aged” subjects. In view of these encouraging early results, we plan further developing our antibody‐based method and aim to eventually present it as a quantitative immunochemical point‐of‐care test that will not require immunofluorescent analysis. Achievement of this goal could facilitate investigation of the beneficial effects of dietary carotenoids on human skin.

## CONFLICT OF INTEREST

Lycotec Ltd developed GA Lycopene, which is evaluated in this study. Ivan M. Petyaev and Nigel H. Kyle are employees of Lycotec; Dmitry V. Pristensky, Marina P. Chernyshova, Marina V. Lozbiakova, and Yuriy K. Bashmakov are independent scientists who collaborate with, but are not, and have never been employees of Lycotec; Natalya E. Chalyk, Victor A. Klochkov, Victoria V. Blinova, Tatiana M. Bogdanova, Alexei A. Iljin, Larisa S. Sulkovskaya and are employees of the Institute of Cardiology in Saratov, Russian Federation; Elena Y. Morgunova, Nailya A. Zigangirova and Valeriy V. Tsibezov are employees of the Gamaleya Research Institute of Epidemiology and Microbiology in Moscow, Russian Federation. There have never been any financial relationships between Lycotec and these collaborating organisations.
